# A 9-year-old Korean girl with Fontaine progeroid syndrome: a case report with further phenotypical delineation and description of clinical course during long-term follow-up

**DOI:** 10.1186/s12881-019-0921-9

**Published:** 2019-11-27

**Authors:** Jaehui Ryu, Jung Min Ko, Choong-Ho Shin

**Affiliations:** Department of Pediatrics, Seoul National University College of Medicine, Seoul National University Children’s Hospital, 101 Daehak-ro, Jongno-gu, Seoul, 03080 South Korea

**Keywords:** Fontaine progeroid syndrome, Gorlin, Chaudhry, Moss syndrome, Fontaine, Farriaux syndrome, S*LC25A24*

## Abstract

**Background:**

Gorlin–Chaudhry–Moss syndrome (GCMS) and Fontaine–Farriaux syndrome (FFS) are extremely rare genetic disorders that share similar clinical manifestations. Because a de novo missense mutation of the solute carrier family 25 member 24 (*SLC25A24*) gene was suggested to be the common genetic basis of both syndromes, it has been proposed recently that they be integrated into a single disorder under the name of Fontaine progeroid syndrome (FPS).

**Case presentation:**

A 9-year-old Korean girl presented with typical clinical features of FPS. She had generalized loose skin with decreased subcutaneous fat, skin wrinkling on the forehead and limbs, skull deformities and a peculiar facial appearance with microphthalmia and midface hypoplasia, anomalies of the digits and nails, a large umbilical hernia and a nearly normal developmental outcome. She exhibited prenatal and postnatal growth retardation together with short stature, and records showed that her height and weight were invariably under − 2.0 SD from birth to the age of 10 years. *SLC25A24* analysis revealed a heterozygous mutation reported previously, NM_013386:c.650G > A, p.[Arg217His]. After screening her family for the identified mutation, she was confirmed as being a de novo case of FPS caused by an *SLC25A24* mutation.

**Conclusion:**

We describe a Korean girl with typical clinical findings of FPS and a de novo mutation in *SLC25A24*, as well as 10 years of clinical follow-up, including growth and developmental achievements.

## Background

Gorlin–Chaudhry–Moss syndrome (GCMS) was first described in 1960 in two girls who had clinical manifestations of craniosynostosis, midface hypoplasia, oligodontia, hypertrichosis, patent ductus arteriosus, anomalies of the eyes and hypoplasia of the labia majora [1]. Since then, additional cases with a similar phenotypic spectrum have been reported [2–4] and it has been suggested that GCMS might be inherited in an autosomal recessive or X-linked dominant pattern with male lethality because all reported subjects were females. Fontaine–Farriaux syndrome (FFS) was independently reported in 1977 in a patient with brachycephaly, anonychia, abdominal muscle hypoplasia and central nervous system anomalies who died at the age of 3 years [5]. Several additional cases of FFS were reported, but all of these patients died before 7 months of age. Even though FFS shares features with GCMS, it was considered to be a separate entity because patients with FFS included both males and females and all died at a younger age than those with GCMS [6–8].

However, with the introduction of next-generation sequencing technologies, including trio exome sequencing and whole-genome sequencing, de novo missense mutations in the hot spot of *SLC25A24* were suggested as the molecular aetiology of both GCMS and FFS by two independent groups in 2017 [9, 10]. Thus, the inheritance pattern of GCMS and FFS could be considered as being autosomal dominant. Because GCMS and FFS share similar clinical presentations and genetic aetiology, Writzl et al. first suggested integrating the two syndromes under the name of Fontaine progeroid syndrome (FPS) because of their patients, who were originally diagnosed as having FFS [10]. The syndromes are now considered as the same disorder on OMIM, under FPS [11], with a wide spectrum of clinical manifestations and severity. However, FPS is extremely rare, with an estimated prevalence of less than 1 in 1,000,000, and only 10 cases (three males and seven females) reported to date [12].

We report a Korean girl with a de novo mutation, NM_013386:c.650G > A, p.[Arg217His], in *SLC25A24* and typical clinical findings of FPS. We also summarize the clinical course of her disease over 10 years.

## Case presentation

The patient was born at 31 weeks and 6 days of gestational age to unrelated healthy parents. No abnormality was found on prenatal sonographic screening and the pregnancy was uneventful until oligohydramnios was observed at 26 weeks, which led to delivery at a birth weight of 1.08 kg (− 1.8 SD) by emergency caesarean section. She was transferred to the neonatal intensive care unit after birth and remained in care for 3 months.

Physical examination showed that she had generalized loose skin, hypertrichosis and an umbilical hernia. She had a dysmorphic face with epicanthus inversus, depressed nasal bridge and low hairline. A congenital skull defect with anterior fontanelle widening and a softened occipital and parietal calvarium were observed. In addition, she had hypoplastic nails and syndactyly of the left 4th and 5th fingers. Based on her clinical phenotype, cutis laxa was initially suspected and conventional karyotyping and direct sequencing of *ATP6V0A2* were performed. However, the results of these analyses were normal.

At the age of 4 months, she visited our hospital for the evaluation of multiple congenital malformations, including congenital skull deformity and hypoplasia of fingers and nails. At that time, her head circumference was 34.3 cm (− 3.8 SD) and radiologic and sonographic findings showed interparietal widening and mild dilatation of the lateral ventricles. Simple radiographs of both hands revealed the absence of the distal phalanx in the 5th fingers and hypoplastic distal phalanges of both sets of 2nd to 4th fingers. Pure tone audiometry revealed mild conductive hearing loss. However, magnetic resonance imaging of the brain and orbit did not find any abnormalities, including the previously noted mild lateral ventricular dilatation. Abdominal sonography and echocardiography showed no structural anomalies, with the exception of a large umbilical hernia.

At the age of 4 years, she underwent a series of surgical procedures, including division of syndactyly of the left 4th and 5th fingers, entropion repair of both upper eyelids and repair of the umbilical hernia. She needed regular ophthalmologic follow-up because of esotropia and severe hyperopia. Her early psychomotor development was delayed as she sat unsupported at 18 months, spoke several words at 24 months and walked independently when she was 36 months old. However, catch-up development allowed her to reach the level of her peers and she is now doing well in school.

**Fig. 1:**
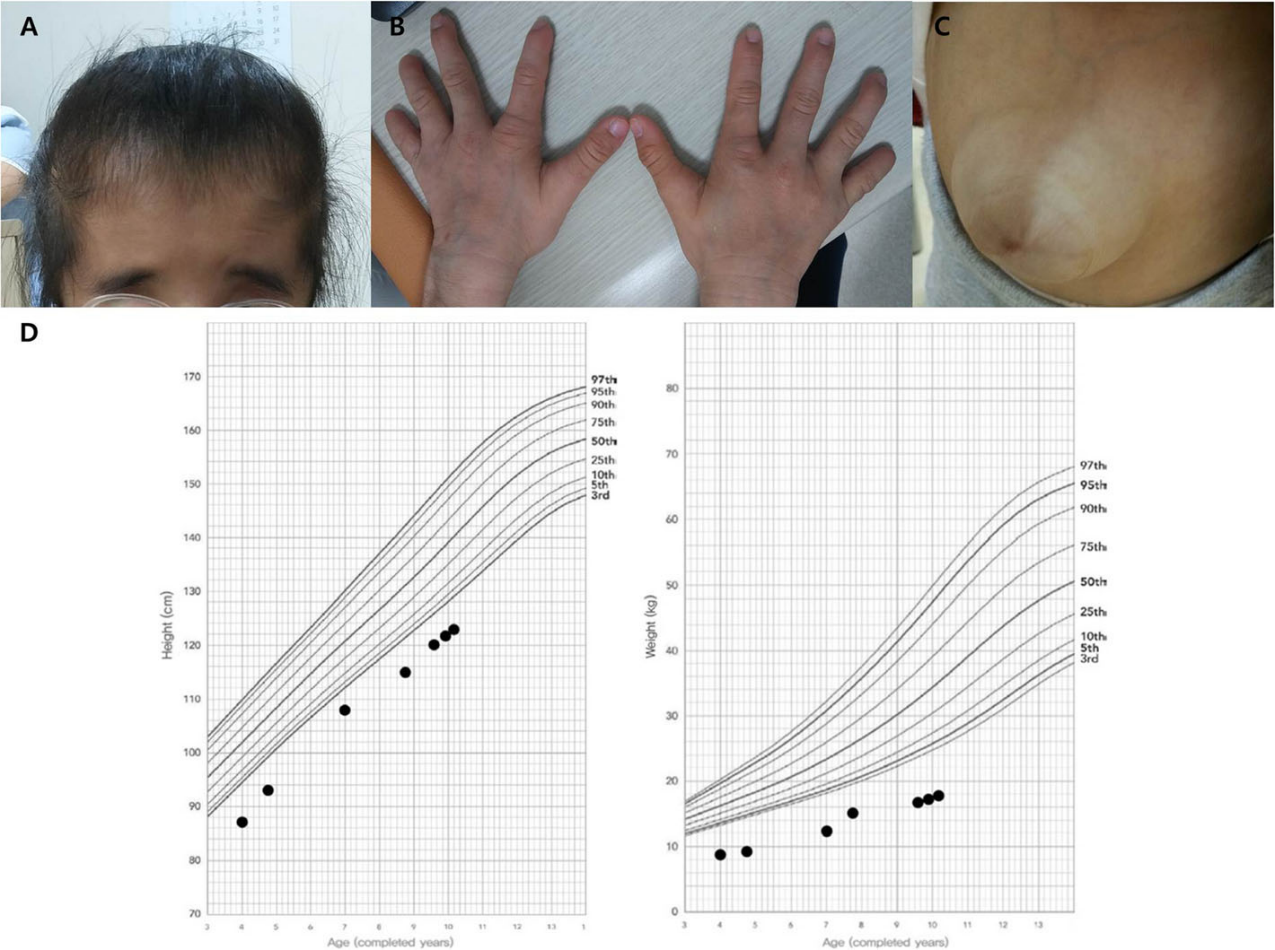
Clinical features of the case reported here. **a**: The characteristic facial appearance included hypertrichosis, frontal bossing, and a low hairline. **b**: Hypoplastic distal phalanges and anonychia are noted in the left 4th and both 5th fingers. **c**: The patient underwent repair of a large umbilical hernia. **d**: The height and weight up to 10 years of age are plotted against a Korean national growth chart [13]. The square markers indicate the data pertaining to our patient

At the age of 8 years, she visited the paediatric genetics clinic of our institution for the identification of the underlying genetic aetiology of her dysmorphic features. Her height, weight and head circumference were 115.0 cm (− 3.0 SD), 15.0 kg (− 4.9 SD) and 48.5 cm (− 2.0 SD), respectively. She had progeroid features with loose skin and wrinkling on the forehead and limbs, as well as generalized hypertrichosis, especially on the forehead and back. A dysmorphic facial appearance with microphthalmia, epicanthus inversus, midface hypoplasia, narrow forehead and low hair line (Fig. 1a), and dysplastic ears with a small and wrinkled morphology were noted. She also had hypoplastic nails on both 5th fingers and left 4th finger (Fig. 1b). The umbilical hernia (Fig. 1c) recurred despite a previous repair operation. Orodental examination revealed a bifid uvula and oligodontia with malocclusion. Although her growth velocity was within the normal range, her height and weight profiles remained under − 2.0 SD (Fig. 1d). A growth hormone (GH) stimulation test and a low-dose adrenocorticotropin stimulation test were performed to rule out pituitary and hypothalamic dysfunction; the results of both tests were normal. At the age of 9 years, a dilated ascending aorta (2.7 cm, + 5.0 SD) was detected using chest computed tomography during pre-operative evaluation of esotropia repair.

**Fig. 2:**
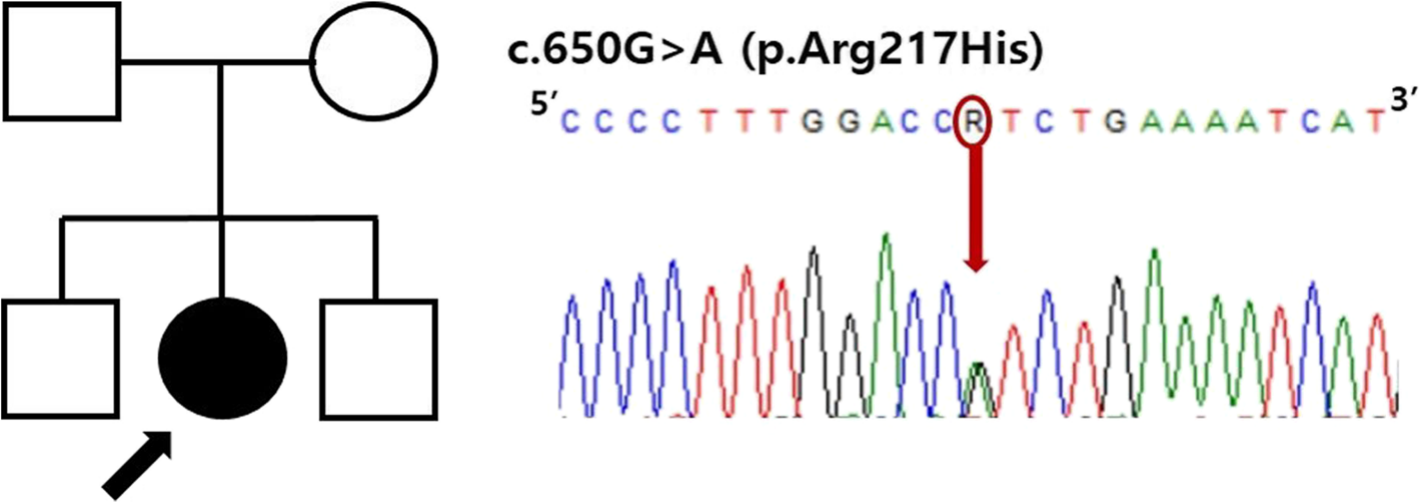
Sanger-sequencing chromatogram of our patient. Partial sequencing of the cloning allele of *SLC25A24* showed the presence of a heterozygous missense mutation, NM_013386:c.650G > A, p.[Arg217His], in exon 5

Based on the medical history and physical examinations, the girl was suspected of having FPS. A chromosomal microarray analysis and direct sequencing of the mutation hot spot of *SLC25A24* for FPS were performed. Written informed consent was obtained from the patient and her parents, and the Institutional Review Board of the Seoul National University Hospital approved this study. The chromosomal microarray analysis did not identify any pathologic copy number variations (Additional file 1), while analysis of exon 5 of *SLC25A24* revealed a heterozygous mutation reported previously, NM_013386:c.650G > A, p.[Arg217His] (Fig. 2). Because her parents and two brothers did not carry this mutation, our patient was confirmed as being a de novo case of FPS caused by an *SLC25A24* mutation.

## Discussion and conclusion

The *SLC25A24* gene encodes a mitochondrial inner-membrane transporter, ATP-Mg/Pi carrier, which consists of six helical structures. Among the *SLC25A24* protein structures, Arg217 plays an important role in maintaining four strong hydrogen bonds between helices 2 and 3 [14]. Patients with FPS reported previously had a concurrent mutation on Arg217, either p.[Arg217His] or p.[Arg217Cys] [9–11]. This leads to distortion of the structure of the ATP-Mg/Pi carrier by narrowing the substrate cavity and disturbing the entry of nucleosides into the carrier. If nucleoside entry is disturbed and an adequate adenine level is not achieved in the mitochondrial matrix, mitochondria undergo calcium overload, which triggers mitochondrial permeability transition mediated by mitochondrial permeability transition pores; this leads to disruption of the mitochondrial membrane potential, mitochondrial swelling and, finally, to cell death [14]. Accordingly, cells with mutated *SLC25A24* are more susceptible to death; this mechanism contributes to several clinical features, such as lipoatrophy or skeletal underdevelopment, which can cause a progeroid appearance. However, because this mechanism cannot explain phenotypes such as microphthalmia or hypertrichosis, the actual mechanism underlying this condition must be determined.

After *SLC25A24* was reported as the common genetic basis of GCMS and FFS, both syndromes were integrated into a single disorder under the name of FPS [9–11]. The major clinical presentations of FPS, as documented in Table 1, include a generally aged appearance with loose or wrinkled skin, short stature, hypertrichosis, skull deformities with craniosynostosis or brachycephaly and a characteristic facial appearance of depressed nasal bridge, low hairline and microphthalmia. These patients also exhibit digit and nail anomalies, cardiovascular abnormalities, umbilical hernia, a hypoplastic genital system and normal or nearly normal developmental outcomes. However, FPS was known as two separate entities, GCMS and FFS, because of major differences between them, i.e. patients with GCMS were mostly female and most patients with FFS died before 1 year of age. To date, a total of 11 patients, three males and eight females, were genetically confirmed as having FPS. Two out of the three male patients died before 12 months of age, and there is only one case of a surviving male. In contrast, only one patient died before 12 months of age and two patients who died at 18 and 20 months of age, among the eight female patients. Our patient presented with typical clinical manifestations of FPS and is more appropriately sub-classified as GCMS, rather than FFS, based on conventional criteria, because she is female and has survived at least until the age of 10 years (Table 2). Further studies are necessary to clarify the basis of the higher prevalence of the *SLC25A24* mutation in females and the higher mortality rate in young males with FPS.

**Table 1:** Clinical manifestations in our patient and previously reported cases of FPS

	Our study	Individual 1^a^	Individual 2^a^	Individual 3^a^	Individual 4^a^	Individual 5^a^	Individual 6^b^	Individual 7^b^	Individual 8^b^	Individual 9^b^	Individual 10^c^	Total
*SLC25A24* mutation	c.650G > A (p.Arg217His)	c.650G > A (p.Arg217His)	c.650G > A (p.Arg217His)	c.650G > A (p.Arg217His)	c.650G > A (p.Arg217His)	c.649G > T (p.Arg217Cys)	c.650G > A (p.Arg217His)	c.650G > A (p.Arg217His)	c.649G > T (p.Arg217Cys)	c.650G > A (p.Arg217His)	c.650G > A (p.Arg217His)	c.650G > A: c.649G > T = 9: 2
Mutation type	heterozygous, de novo	heterozygous, de novo	heterozygous, de novo	heterozygous, de novo	heterozygous, de novo	heterozygous, de novo	heterozygous, de novo	heterozygous, de novo	heterozygous, de novo	heterozygous, de novo	heterozygous, de novo	
Sub-clinical classification	GCMS	GCMS	GCMS	GCMS	GCMS	GCMS	FFS	FFS	FFS	FFS	FFS	
Sex	female	female	female	female	female	female	male	female	female	male	male	M: F = 3: 8
Ethnicity	Korean	Polish	Hungarian	German	Turkish	Northern European	Slovene	French	Spanish	Italian	Spanish	
Age at last exam (or age at death)	9 years	5.5 years	7 years	5 years	(20 months)	14 years	(6 months)	(7 months)	(7 h)	(20 h)	15 years	9.3 ± 4.3 years (6.6 ± 8.2 months)
Gestational age	32 weeks	39 weeks	36 weeks	38 weeks	39 weeks	37 weeks	34 weeks	38 weeks	35 weeks	32 weeks	35 weeks	35.9 ± 2.5 weeks
IUGR	+	+	–	+	+	+	+	+	+	+	+	9/11 (82%)
Birth weight	1080 g	2200 g	2225 g	1600 g	1700 g	1722 g	1390 g	1700 g	800 g	866 g	1450 g	1521.2 ± 472.5 g
HC at birth	NK	28 cm	NK	29 cm	29.4 cm	NK	29 cm	28.5 cm	25.9 cm	23 cm	28 cm	27.6 ± 2.1 cm
Aged appearance	+	+	+	+	+	+	+	+	+	+	+	11/11 (100%)
Short stature	+	+	–	+	+	+	+	+	+	+	+	9/11 (82%)
Wrinkled and translucent skin	+	+	+	+	+	+	+	+	+	+	+	11/11 (100%)
Hypertrichosis	+	+	+	+	+	+	NK	NK	NK	+	+	8/8 (100%)
Coronal craniosynostosis	+	+	+	+	+	NK	+	NK	NK	+	+	8/8 (100%)
Brachycephaly	+	+	+	+	+	+	NK	NK	+	NK	–	7/8 (88%)
Large anterior fontanel	+	+	–	+	+	–	+	+	+	+	+	9/11 (82%)
Triangular face	+	+	NK	–	+	NK	+	+	+	+	+	8/9 (89%)
Midface hypoplasia	+	+	+	+	+	+	–	–	NK	NK	+	7/9 (78%)
Depressed nasal bridge	+	+	+	+	+	+	+	+	+	+	–	10/11 (91%)
Low hairlines	+	+	+	+	+	+	+	+	+	+	+	11/11 (100%)
Low set, dysplastic ears	+	–	+	+	+	+	+	+	+	+	+	10/11 (91%)
Micropthalmia	+	+	+	+	+	+	+	+	+	+	+	10/11 (91%)
Downslanting palpebral fissure	+	+	+	+	+	+	NK	NK	NK	NK	–	6/7 (86%)
Prognathia	+	+	+	–	+	+	NK	NK	NK	NK	+	6/7 (86%)
Short distal phalanges	+	–	–	+	+	+	+	+	+	+	+	9/11 (82%)
Hypoplastic nails	+	+	–	+	+	+	+	+	+	+	+	10/11 (91%)
Syndactyly	+	–	+	+	–	+	NK	NK	NK	–	–	4/8 (50%)
Cardiovascular abnormalities	+	NK	NK	+	+	+	+	+	–	–	+	7/8 (75%)
Hypoplastic external genitalia	–	+	+	+	+	+	–	NK	+	+	+	8/10 (89%)
Cryptorchidism	NA	NA	NA	NA	NA	NA	+	NA	NA	+	+	3/3 (100%)
Umbilical hernia	+	+	–	+	+	+	+	+	–	+	+	9/11 (82%)
Conductive hearing loss	+	–	–	+	+	+	NA	NA	NA	NA	NK	4/6 (67%)
Normal developmental outcome	+	+	+	+^d^	+	+^d^	NA	+	NA	NA	+	8/8 (100%)

*Abbreviations*: +, present; −, not present; *HC* Head circumference, *IUGR* Intrauterine growth retardation, *NA* Not applicable, *NK* Not known;

^a^Ehmke et al. [9]

^b^Writzl et al. [10]

^C^Rodriguez et al. (2018) [12]

^d^Delayed motor development due to muscle weakness

**Table 2:** Demographic information of all reported cases of FPS-related syndromes

		Sex	Age at last exam	Age of death	Cause of death	Gene confirmation of *SLC25A24* mutations
Gorlin-Chaudhry-Moss syndrome (GCMS)	Gorlin et al. (1960) [1]	F	10 years			
		F	8 years			
	Ippel et al. (1992) [2]	F	4 years			
		F	33 years			
	Aravena et al. (2011) [3]	F		5 months	pneumonia	
		F		18 months	pneumonia	
	Rosti et al. (2013) [4]	F	4 years			
	Ehmke et al. (2017) [9]	F	5.5 years			+
		F	7 years			+
		F	5 years			+
		F		20 months	urinary tract infection	+
		F	14 years			+
	Our patient (2018)	F	9 years			+
Fontaine-Farriaux syndrome (FFS)	Fontaine et al. (1977) [5]	M		3 months	unknown	
	Faivre et al. (1999) [6]	F		7 months	sepsis	+
	Rodriguez et al. (1999) [7]	F		7 h	respiratory distress	+
	Castori et al. (2009) [8]	M		20 h	respiratory distress	+
	Writzl et al. (2017) [10]	M		6 months	cardiorespiratory arrest	+
	Rodriguez et al. (2018) [12]	M	15 years			+
Petty-Laxova-Wiedemann syndrome (PLWS)	Petty et al. (1990) [15]	F	45 years			
		F	5 years			
	Delgado et al. (2009) [16]	M	10 years			
	Braddock et al. (2010) [17]	F		7 months	respiratory failure	
		M		23 months	sepsis	

Another progeroid syndrome, Petty–Laxova–Wiedemann syndrome (PLWS), also presents with the typical characteristics of FPS, including prenatal growth restriction and subsequent short stature, decreased subcutaneous fat, coronal synostosis, umbilical hernia at birth, short digital phalanges and normal development [15–17]. However, genetic confirmation has not been performed in any of the reported patients with PLWS. If PLWS survivors are available, we suggest verifying whether they harbour the same mutation in *SLC25A24*. In this study, we follow up the height and weight of our patient from infancy to 10 years of age and plotted them on a growth chart (Fig. 1d, Table 3). Considering that she was born pre-term and was small for gestational age newborn, the height and body weight were invariably under − 2.0 SD compared with corrected age- and sex-matched controls. Her height velocity was within the normal range and hormonal evaluations did not show any deficiencies.

**Table 3:** Heights, weights and their standard deviations according to ages

Age	Height (SD)		Weight (SD)	
At birth	Unknown		1.1 kg	(−3.8)
1 year 5 months	65.4 cm	(−4.4)	4.4 kg	(−10.7)
4 years	87.1 cm	(−3.3)	8.5 kg	(−6.9)
4 years 9 months	93.0 cm	(− 3.1)	9.0 kg	(−7.6)
7 years	108.3 cm	(−2.7)	12.6 kg	(−5.6)
8 years 9 months	115.0 cm	(− 3.0)	15.0 kg	(− 4.9)
9 years 7 months	120.4 cm	(− 2.6)	16.4 kg	(− 4.6)
9 years 11 months	121.8 cm	(− 2.5)	16.9 kg	(− 4.6)
10 years 2 months	123.0 cm	(− 2.5)	17.5 kg	(− 4.5)

Because the majority of patients with FPS are expected to have a nearly normal lifespan, the improvement of their quality of life by correcting deformities and managing medical issues is important. One report described the surgical correction of craniofacial malformations in a 7-year-old patient with GCMS using internal distraction devices [18]. Another report described a 33-year-old GCMS patient who presented with an irregular menstrual cycle and infertility [2]. It is important to monitor secondary sexual development and reproductive ability in, and provide genetic counselling to, these patients. Furthermore, because congenital cardiac anomalies are often observed in patients with FPS [13], regular medical surveillance of cardiovascular complications is essential.

This report presented a Korean case of FPS with genetic confirmation, as well as long-term growth follow up. We monitored serially the growth profile of our patient and found that her short stature was not caused by GH deficiency. We also organized chronologically the demographic information of related syndromes, including GCMS, FFS and PLWS. Even though FPS is an extremely rare disorder, efforts aimed at providing medical support based on its pathophysiology, as well as investigating unresolved questions, including the mechanism underlying the intersexual differences observed, should continue.

## Supplementary information

**Additional file 1.** Detail and data of chromosomal microarray testing including non-pathogenic CNVs.

## Data Availability

The datasets generated during and/or analyzed during the current study are not publicly available because it is possible that individual privacy could be compromised. However, datasets are available from the corresponding author on reasonable request.

## Abbreviations

Arg: Arginine
Cys: Cysteine
FFS: Fontaine–Farriaux syndrome
FPS: Fontaine progeroid syndrome
GCMS: Gorlin–Chaudhry–Moss syndrome
GH: Growth hormone
His: Histidine
NA: Not applicable
NICU: Neonatal intensive care unit
NK: Not known
PLWS: Petty–Laxova–Wiedemann syndrome
SLC25A24: Solute carrier family 25 member 24
