# Supplementary material for: A 9-year-old Korean girl with Fontaine progeroid syndrome: a case report with further phenotypical delineation and description of clinical course during long-term follow-up
Source: BMC Med Genet. 2019 Nov 27;20:188. doi: 10.1186/s12881-019-0921-9 (PMC6882017; doi:10.1186/s12881-019-0921-9)
Supplement: Supplementary file 1 — Additional file 1. Detail and data of chromosomal microarray testing including non-pathogenic CNVs. [file 12881_2019_921_MOESM1_ESM.docx]

Additional file 1

1. Detail of chromosomal microarray testing

Chromosomal microarray testing was conducted using Agilent Human Genome oligonucleotide comparative genomic hybridization (CGH) microarray 4 x 180K (Agilent Technologies, Santa Clara, CA, USA) with 13 kb overall median probe spacing. Genomic DNA was labeled and hybridized to the array, according to the manufacturer’s protocol for Oligonucleotide Array-Based CGH for Genomic DNA Analysis (version 6.2; Agilent Technologies). A DNA reference sample (male or female human genomic DNA; Promega, Madison, WI, USA) was used. The slide was scanned on a microarray scanner (G2565CA; Agilent Technologies). Data were extracted from a *.tif image using Agilent Feature Extraction software (version 10.7.3.1) and analyzed with Genomic Workbench software (version 7.0.4.0, Agilent Technologies). The local background was subtracted from the median intensities of the Cy3 and Cy5 channels. The log2 patient-to-reference ratio was calculated for each spot and normalized to the median of the ratio of all chromosomes. All CNVs were called and based on human assembly GRCh37 (hg19).

1. Data of chromosomal microarray including non-pathogenic CNVs

| Event No. | Chr | Cytoband | #Probes | Amp/Del | P-value | Annotations |
| --- | --- | --- | --- | --- | --- | --- |
| 1 | Chr1:248727929-248808452 | q44 | 7 | 0.558481 | 1.10E-11 | OR2T34, OR2T10, OR2T11… |
| 2 | Chr4:69392545-69462438 | q13.2 | 6 | -1.916967 | 4.13E-58 | UGT2B17, UGT2B15 |
| 3 | Chr7:38296176-38352444 | p14.1 | 6 | -0.572473 | 3.40E-11 | TARP |
| 4 | Chr8:39237438-39345479 | p11.22 | 10 | 0.469793 | 8.95E-13 | ADAM5P, ADAM3A |
| 5 | Chr11:7817491-7826827 | p15.4 | 3 | -0.936854 | 4.25E-13 | OR5P2 |
| 6 | Chr11:55377910-55450788 | q11 | 8 | -0.607647 | 2.28E-15 | OR4P4, OR4S2, OR4C6 |
| 7 | Chr12:9637323-9713425 | p13.31 | 5 | 0.776446 | 1.22E-15 |  |
| 8 | Chr12:11221333-11256467 | p13.2 | 3 | 1.252332 | 2.18-17 | PRR4, PRH1, TAS2R43 |
| 9 | Chr13:63361547-63629173 | q21.31 | 8 | -0.985970 | 8.67E-35 |  |
| 10 | Chr14:22554846-22976316 | q11.2 | 38 | -0.498893 | 2.07E-47 |  |
| 11 | Chr14:106531557-106559103 | q32.33 | 3 | -2.248177 | 6.60E-30 |  |
| 12 | Chr15:20481702-22558756 | q11.1-q11.2 | 34 | 0.441264 | 2.36E-34 | GOLGA6L6, GOLGA8C, BCL8… |
| 13 | Chr16:3219476-3237685 | p13.3 | 3 | -0.977046 | 2.16E-14 |  |
| 14 | Chr16:33462958-33625989 | p11.2 | 7 | -0.517814 | 1.96E-10 |  |
| 15 | Chr22:24347959-24390254 | q11.23 | 5 | -2.497861 | 2.75E-62 | LOC391322, GSTT1, GSTTP2 |

Amp, Amplification; Del, Deletion
